# Clinical and metagenomic characterization of HHV-6 versus HHV-7 encephalitis in immunocompetent children

**DOI:** 10.3389/fcimb.2026.1909287

**Published:** 2026-10-01

**Authors:** Erhu Wei, Panpan Shang, Fengyan Li, Kunli Yan, Mengjiao Zhang, Xiao Fang, Peisheng Jia, Jiandong Wang, Jing Zhao, Na Li, Huaili Wang, Nanchang Xie

**Affiliations:** 1 Pediatric Intensive Care Unit, The First Affiliated Hospital of Zhengzhou University, Zhengzhou, Henan, China; 2 Department of Pediatrics, Taiyuan Maternal and Child Health Hospital, Taiyuan, Shanxi, China; 3 Pediatric Intensive Care Unit, Nanyang Children’s Medical Center, Nanyang, Henan, China; 4 Department of Pediatrics, Jiyuan People’s Hospital, Jiyuan, Henan, China; 5 Department of Neurology, The First Affiliated Hospital of Zhengzhou University, Zhengzhou, Henan, China

**Keywords:** betaherpesvirus, cerebrospinal fluid, encephalitis, HHV-6, HHV-7, metagenomic next-generation sequencing

## Abstract

**Background:**

Human herpesvirus 6 (HHV-6) and human herpesvirus 7 (HHV-7) are betaherpesviruses that can cause encephalitis/encephalopathy in immunocompetent children. However, early clinical manifestations, routine laboratory tests, and neuroimaging findings often lack specificity, making etiological characterization challenging. This study aimed to characterize the clinical features and metagenomic findings of HHV-6 and HHV-7 encephalitis in immunocompetent children.

**Methods:**

This single-center retrospective cohort study included children (<18 years) with suspected central nervous system infection who underwent lumbar puncture and cerebrospinal fluid (CSF) next-generation sequencing (mNGS) testing at The First Affiliated Hospital of Zhengzhou University between January 2020 and December 2024. Patients with HHV-6 or HHV-7 sequences detected in CSF by mNGS, together with compatible clinical features and exclusion of other more likely etiologies, were included. Demographic characteristics, clinical manifestations, laboratory and CSF findings, neuroimaging features, mNGS results, treatments, and short-term outcomes were analyzed.

**Results:**

A total of 20 children with CSF HHV-6 or HHV-7 sequence detection were included (HHV-6, n=5; HHV-7, n=15). HHV-6 cases mainly occurred in infants and young children, whereas HHV-7 cases were predominantly observed in older children. Fever and seizure-related symptoms were the most common clinical manifestations. Peripheral inflammatory markers, CSF parameters, and neuroimaging findings showed heterogeneous patterns among patients. CSF mNGS detected HHV-6 or HHV-7 sequences in all included cases, with variable sequencing read counts (HHV-6: median 257 reads, range 55–52, 686; HHV-7: median 2, 635 reads, range 457–26, 355). The median turnaround time of mNGS reporting was 1 day (range, 1–2 days). All patients received antiviral therapy, with some receiving adjunctive corticosteroids and/or intravenous immunoglobulin. Short-term outcomes at discharge were generally favorable, and only one patient developed secondary autoimmune encephalitis.

**Conclusions:**

HHV-6 and HHV-7 encephalitis in immunocompetent children share overlapping clinical characteristics and lack specific early laboratory or imaging features. CSF mNGS enabled rapid and unbiased detection of HHV-6 and HHV-7 sequences and provided complementary etiological information when interpreted together with clinical and ancillary findings.

## 1 Introduction

Human herpesvirus 6 (HHV-6) is a neurotropic betaherpesvirus. Primary infection occurs predominantly in infants and young children, and a small proportion may progress to central nervous system (CNS) infection, presenting as encephalitis with seizures and impaired consciousness (Zerr, 2006). Its clinical phenotypes are diverse and lack specificity, making it difficult to distinguish from other viral encephalitides in the early stage (Berzero et al., 2021). Human herpesvirus 7 (HHV-7), also a betaherpesvirus, can likewise cause encephalitis/encephalopathy in immunocompetent children, and viral nucleic acid may be detected in the cerebrospinal fluid (CSF) (Chen et al., 2025; Moppert et al., 2024). In clinical practice, both HHV-6 and HHV-7 associated encephalitis may show non-specific findings on early ancillary tests; conventional etiological testing often relies on targeted screening, which may limit timely etiological clarification.

Metagenomic next-generation sequencing (mNGS) offers unbiased, broad-spectrum pathogen detection and has clinical utility in CNS infections with unclear etiological clues (Chen et al., 2024; Piantadosi et al., 2021). However, comparative evidence for betaherpesvirus encephalitis in immunocompetent children—especially HHV-6 versus HHV-7 remains limited. Therefore, we described and summarized clinical characteristics, ancillary examinations, mNGS findings, treatment regimens, and outcomes in patients with HHV-6 and HHV-7 encephalitis to characterize their clinical and metagenomic features.

## 2 Methods

### 2.1 Study design and participants

This was a single-center retrospective cohort study. We enrolled hospitalized children (<18 years) at The First Affiliated Hospital of Zhengzhou University who underwent lumbar puncture and cerebrospinal fluid (CSF) metagenomic next-generation sequencing (mNGS) testing for suspected central nervous system (CNS) infection (encephalitis/meningitis) between January 2020 and December 2024. Data collected included demographic characteristics (age, sex, season of onset), clinical manifestations (fever, seizures/status epilepticus, altered consciousness, etc.), peripheral blood inflammatory markers, CSF parameters (nucleated cell count, protein, glucose), imaging findings, mNGS results, treatment regimens, and outcomes. This study was approved by the Ethics Committee of The First Affiliated Hospital of Zhengzhou University.

Inclusion criteria: (1) age <18 years; (2) acute or subacute onset (disease course <12 weeks) with CNS involvement manifestations such as fever, seizures, and/or altered mental status; (3) lumbar puncture with CSF mNGS testing completed during hospitalization. Exclusion criteria: (1) a confirmed history or evidence of trauma, poisoning, metabolic disorders, brain tumors, demyelinating diseases, or hereditary neurological disorders; (2) significant immunosuppression (e.g, post-organ transplantation or long-term use of immunosuppressants); (3) Cases with potential CSF coinfection were excluded. Coinfection was defined as the detection of more than one clinically relevant pathogen (including bacteria, viruses, fungi, or parasites) in CSF by mNGS or targeted pathogen testing. All included patients had HHV-6 or HHV-7 identified as the only potential pathogen detected in CSF.

Grouping and definitions: Based on CSF mNGS, cases were categorized into an HHV-6 group and an HHV-7 group. Case attribution was based on an integrated assessment of clinical manifestations, CSF findings, neuroimaging features, and exclusion of other potential etiologies, rather than mNGS positivity alone. HHV-6 or HHV-7 detected by CSF mNGS was considered supportive evidence of potential pathogen involvement when viral sequences showed predominance among detected microorganisms and no alternative pathogen was identified.

Neurological status at discharge was assessed using the Pediatric Cerebral Performance Category Scale (PCPCS), a scale commonly used to evaluate neurological function in children after acute brain injury or neurological disorders, including severe infections. The PCPCS consists of six categories ranging from 1 to 6, with higher scores indicating more severe neurological impairment; a score of 1 represents normal neurological function, whereas a score of 6 indicates death.

### 2.2 mNGS protocol

CSF samples were collected following standard clinical procedures. For each patient, 0.5 mL of CSF was mixed with an equal volume of 0.5 mm zirconia/silica beads (BioSpec, Cat. No. 11079105z) for mechanical disruption. DNA was then extracted using the TIAN-Amp Micro DNA Kit. Library construction involved a series of steps including DNA fragmentation, end-repair, A-tailing, adapter ligation, and PCR amplification. For all five patients suspected of RNA virus-associated CNS infection, reverse transcription and second-strand cDNA synthesis were additionally performed. RNA was extracted from an additional 0.5 mL aliquot of the same CSF sample using the QIAamp Viral RNA Mini Kit. Library quality was assessed using the Agilent 2100 system, and qualified libraries were sequenced on the BGISEQ-100 platform.

Each sequencing batch included a negative control (nuclease-free water processed identically to clinical samples) to monitor potential contamination. Additionally, one sample per batch was run in duplicate to ensure the consistency and reliability of the sequencing process; concordant results were considered as an indicator of successful run quality.

Raw sequencing reads were filtered to remove low-quality sequences and those shorter than 35 base pairs. High-quality reads were aligned to human reference genomes (GRCH38: https://ftp.ncbi.nlm.nih.gov/genomes/all/GCF/000/001/405/GCF_000001405.40_GRCh38.p14/GCF_000001405.40_GRCh38.p14_genomic.fna.gz) using the Burrows-Wheeler Aligner (BWA, v0.7.10) to remove human-derived sequences. Non-human reads were subsequently classified by alignment against an in-house microbial genome database encompassing viral, bacterial, fungal, and parasitic reference sequences. Reference datasets were obtained from the NCBI RefSeq database, including 2, 700 viral genomes, 1, 494 bacterial genomes or scaffolds, 73 human-infecting fungi, and 47 pathogenic parasites. Microbial abundance and genome coverage were calculated using the SoapCoverage tool.

The interpretation of mNGS results followed a multi-step filtering and clinical correlation process:

1. Initial filtering: Only microorganisms with at least three uniquely mapped reads and relative abundance exceeding predefined thresholds (established from background data of healthy control CSF samples) were retained for further analysis. Each microorganism was assigned a taxon-specific cutoff value to minimize false positives.
2. Abundance-based prioritization: Among the remaining candidates, pathogens were prioritized if they exhibited the highest absolute abundance within their genus, or ranked among the top 10 for bacteria, viruses, and parasites — and among the top 20 for fungi and Mycobacterium tuberculosis — in terms of relative abundance.
3. Clinical correlation: Organisms classified as well-recognized causes of CNS infections were directly regarded as likely etiologic agents. For rare or atypical microbes, clinical relevance was assessed in conjunction with the patient’s clinical presentation, CSF parameters, and neuroimaging findings. Organisms lacking clinical correlation were interpreted as non-pathogenic or background contaminants.

All non-human sequencing data generated in this study have been submitted to the China National GeneBank under accession number CNP0000610.

Because this was a retrospective study, predefined organism-specific abundance cutoffs and a dedicated healthy-control CSF background reference were not available. Therefore, mNGS findings were interpreted using a prespecified composite framework integrating non-host read support after host subtraction, within-sample relative abundance rank, batch-level negative-control contamination review, and clinicoradiologic concordance. No single metric alone was considered sufficient for etiologic attribution. For latent herpesviruses, particularly HHV-6 and HHV-7, CSF detection was treated as supportive rather than standalone evidence of causality.

### 2.3 Statistical analysis

Continuous variables were presented as mean ± standard deviation or median (interquartile range), and categorical variables were presented as counts and percentages.

## 3 Results

### 3.1 Inclusion and baseline characteristics

A total of 20 children with betaherpesvirus-associated encephalitis were included, comprising 5 cases in the HHV-6 group and 15 cases in the HHV-7 group. In the HHV-6 group, 3 patients were male (60.0%) and 2 were female (40.0%); in the HHV-7 group, 11 were male (73.3%) and 4 were female (26.7%). The median age was 11 months (IQR, 9–12 months) in the HHV-6 group and 108 months [IQR, 36–120 months] in the HHV-7 group, indicating different age distributions between the two groups. The time from symptom onset to admission was 2.0 days (IQR, 2.0–2.0 days) in the HHV-6 group and 2.0 days (IQR, 1.5–3.0 days) in the HHV-7 group. Regarding seasonality, cases in the HHV-6 group occurred predominantly in summer (3/5), whereas HHV-7 cases were distributed across all seasons (4 in winter, 3 in spring, 5 in summer, and 3 in autumn), as shown in **Table 1**.

**Table 1:** Baseline characteristics and clinical manifestations of children with HHV-6 and HHV-7 encephalitis.

Variable	HHV-6 group (n=5)	HHV-7 group (n=15)
Age, years, median (IQR)	0.92 (0.75–1)	9 (3–10)
Male, n (%)	3 (60.0%)	11 (73.3%)
Time from onset to admission, days, median (IQR)	2 (2–2)	2 (1.5–3)
Fever, n (%)	5 (100.0%)	14 (93.3%)
Seizures, n (%)	4 (80.0%)	10 (66.7%)
Status epilepticus, n (%)	2 (40.0%)	3 (20.0%)
Disturbance of consciousness, n (%)	0 (0.0%)	1 (6.7%)
Headache/vomiting, n (%)	1 (20.0%)	3 (20.0%)
Focal neurological signs, n (%)	0 (0.0%)	2 (13.3%)

### 3.2 Comparison of clinical manifestations

Fever and seizure-related symptoms were the predominant clinical manifestations in both groups. Fever was observed in all patients in the HHV-6 group (5/5, 100%) and in most patients in the HHV-7 group (14/15, 93.3%). Seizures were common in both groups, occurring in 4/5 patients (80.0%) in the HHV-6 group and 10/15 patients (66.7%) in the HHV-7 group. Status epilepticus was observed in 2/5 patients (40.0%) with HHV-6 encephalitis and 3/15 patients (20.0%) with HHV-7 encephalitis.

### 3.3 Comparison of laboratory and CSF findings

The median white blood cell (WBC) count was 7.67 (IQR, 4.99–11.81) ×10^9^/L in the HHV-6 group and 6.09 (IQR, 5.21–9.54) ×10^9^/L in the HHV-7 group. The neutrophil percentage was 44.1% (IQR, 27.4%–74.4%) and 64.9% (IQR, 50.3%–86.1%), respectively, whereas the lymphocyte percentage was 48.6% (IQR, 16.9%–61.0%) and 26.5% (IQR, 8.7%–43.3%).The median C-reactive protein (CRP) levels were 2.88 (IQR, 0.90–3.30) mg/L in the HHV-6 group and 1.71 (IQR, 0.77–9.12) mg/L in the HHV-7 group. The corresponding procalcitonin (PCT) levels were 0.187 (IQR, 0.059–0.200) ng/mL and 0.106 (IQR, 0.072–0.491) ng/mL, respectively. interleukin-6 (IL-6) levels were 5.95 (IQR, 1.75–27.60) in the HHV-6 group and 7.69 (IQR, 3.84–21.47) in the HHV-7 group.

The median CSF nucleated cell count was 1 (IQR, 0–1) ×10^6^/L in the HHV-6 group and 25 (IQR, 6–46) ×10^6^/L in the HHV-7 group. Notably, one patient in the HHV-6 group exhibited a markedly elevated CSF nucleated cell count (maximum 711 ×10^6^/L), whereas most other patients had relatively low CSF cell counts. CSF total protein levels were 217 (IQR, 203–236) mg/L in the HHV-6 group and 254 (IQR, 156–349) mg/L in the HHV-7 group. CSF chloride concentrations were 119.0 (IQR, 118.2–120.4) mmol/L and 120.0 (IQR, 117.8–124.0) mmol/L, respectively. CSF glucose levels were 3.73 (IQR, 3.41–4.02) mmol/L and 3.59 (IQR, 3.40–4.12) mmol/L, respectively, as shown in **Table 2**.

**Table 2:** Laboratory and CSF characteristics of children with HHV-6 and HHV-7 encephalitis.

Parameter	HHV-6 group (n=5)	HHV-7 group (n=15)
Peripheral WBC (×10^9/L) (IQR)	7.67 (4.99–11.81)	6.09 (5.21–9.54)
CRP (mg/L) (IQR)	2.888 (0.90–3.30)	1.71 (0.77–9.12)
PCT (ng/mL) (IQR)	0.187 (0.059–0.200)	0.106 (0.072–0.491)
CSF WBC (×10^6/L) (IQR)	1 (0–1)	25 (6–46)
CSF protein (mg/L) (IQR)	217 (203–236)	254 (156–349)
CSF glucose (mmol/L) (IQR)	3.73 (3.41–4.02)	3.59 (3.40–4.12)
CSF chloride (mmol/L) (IQR)	119.0 (118.2–120.4)	120.0 (117.8–124.0)

### 3.4 Neuroimaging findings

All five patients in the HHV-6 group underwent brain MRI. Four (80.0%) showed no obvious abnormalities, and one (20.0%) had periventricular signal changes, considered consistent with incomplete myelination. All 15 patients in the HHV-7 group underwent neuroimaging; 11 (73.3%) had no obvious abnormalities and 4 (26.7%) showed abnormal signals. Abnormalities in the HHV-7 group mainly consisted of scattered, mildly prolonged T2 signals in the cortex/subcortical regions and white matter, involving cortical areas such as the parietal, frontal, and temporal lobes. In some cases, lesions were multifocal and patchy, with possible involvement of periventricular regions, basal ganglia, and thalamus.

### 3.5 CSF mNGS results and time to reporting

All 20 children underwent CSF mNGS testing, and betaherpesvirus-related sequences were detected in all cases. In the HHV-6 group (n=5), HHV-6 sequences were identified, with a median read count of 257 (IQR, 74–1027; range, 55–52, 686). In the HHV-7 group (n=15), HHV-7 sequences were detected in all cases, with a median read count of 2, 635 (IQR, 896.5–6, 136.5; range, 457–26, 355).

The median turnaround time from CSF sample collection to mNGS report was 1 day (range, 1–2 days) in the overall cohort.

### 3.6 Treatment and outcomes

All patients received antiviral therapy. In the HHV-6 group, ganciclovir was the main antiviral agent (3/5, 60.0%), while the remaining two cases received penciclovir (2/5, 40.0%). In the HHV-7 group, 7/15 (46.7%) received ganciclovir, 5/15 (33.3%) received acyclovir, and 3/15 (20.0%) received penciclovir.

For immunomodulatory therapy, 1 patient in the HHV-6 group received corticosteroids (20.0%), and 1 received combined intravenous immunoglobulin (IVIG) (20.0%). In the HHV-7 group, 4 patients received corticosteroids (26.7%), and 2 received combined IVIG (13.3%).

At discharge, all patients in the HHV-6 group had a PCPCS score of 1 (5/5, 100%), indicating overall favorable outcomes. In the HHV-7 group, 14/15 (93.3%) had a PCPCS score of 1, and 1/15 (6.7%) had a PCPCS score of 3. Regarding complications, 2 patients (40.0%) in the HHV-6 group had seizure-related complications and 1 (20.0%) developed peripheral facial nerve palsy later in the disease course. In the HHV-7 group, only one patient (6.7%) developed secondary autoimmune encephalitis.

## 4 Discussion

HHV-6 is widely distributed worldwide, with geographic variation in infection rates; the reported prevalence is approximately 60%–90% (Krueger and Ablashi, 2003). Seropositivity in healthy adults can exceed 95%, and antibody titers gradually decline with age (Gough et al., 2026). Although HHV-6 infection is not uncommon in immunocompetent children, most cases are subclinical or mild, and only a subset present with classic exanthem subitum; clinical phenotypes may vary depending on individual immune status (Zerr et al., 2005). In contrast, in populations with primary or secondary cellular immunodeficiency, latent HHV-6 is more prone to reactivate and cause fever, rash, hepatitis, and CNS involvement; previous reports also suggest high reactivation rates in hematopoietic stem cell and solid organ transplant recipients (Tunkel et al., 2008). However, most investigators consider HHV-6 encephalitis relatively uncommon in immunocompetent children, and its pathogenicity and causal attribution still require pathogen evidence integrated with clinical consistency (Berzero et al., 2021; Webb et al., 2024). In line with our comparative results, HHV-6 cases clustered in infants and young children, while HHV-7 cases were more frequent in older children. This age stratification may serve as a relatively stable clinical clue for differentiation, but it cannot replace etiological confirmation.

In this cohort of 20 pediatric patients with betaherpesvirus-associated encephalitis (5 HHV-6 and 15 HHV-7 cases), clinical manifestations and ancillary examination findings showed substantial overlap between HHV-6 and HHV-7 encephalitis. Fever and seizure-related symptoms were the predominant clinical features, whereas routine laboratory parameters generally lacked pathogen specificity.

Previous studies have reported that HHV-6 encephalitis may involve the hippocampus and temporal lobes, sometimes extending to the parahippocampal gyrus or amygdala, and clinically manifesting as an “acute hippocampal dysfunction” phenotype characterized by behavioral changes, confusion, memory impairment, and sleep disturbances (Kawamura et al., 2022; Vu et al., 2007). However, in our HHV-6 cohort, brain MRI did not demonstrate these typical involvement patterns. Even when seizure-related complications occurred, MRI was largely normal or showed only non-specific changes. This suggests that imaging manifestations of HHV-6 encephalitis may be unstable and lack clear specificity, and therefore cannot serve as a definitive diagnostic basis (Noguchi et al., 2006; Sadighi et al., 2015).

Traditional diagnosis of viral encephalitis relies mainly on virological assays, serology, and molecular testing (Debiasi and Tyler, 2004; Tunkel et al., 2008). Although isolation of live virus from CSF or brain tissue is regarded as a “gold standard, “ it is technically demanding, time-consuming, and often limited in sensitivity, making it impractical for rapid clinical decision-making (Xu et al., 2023). Serologic testing typically becomes positive later in the course and may be affected by cross-reactivity, limiting its value for acute confirmation (Kahraman et al., 2017). Molecular methods such as PCR are sensitive and rapid, but they are usually targeted, covering a limited number of pathogens per test; thus, they remain constrained when the pathogen spectrum is broad or co-infection is suspected (Houlihan et al., 2019; Olie et al., 2024).

Previous studies have reported that HHV-6 and HHV-7 encephalitis may present with diverse and non-specific clinical manifestations, and conventional laboratory and pathogen detection approaches may not always provide sufficient etiological information (Benoit et al., 2024; Chen et al., 2024). Consistent with these observations, our findings suggest that relying solely on conventional clinical features and routine pathogen testing may pose challenges in the identification of HHV-6 and HHV-7 encephalitis, may provide additional etiological information to support clinical assessment. Against this backdrop, mNGS—an unbiased, high-throughput, broad-spectrum detection technique—can simultaneously interrogate viral, bacterial, fungal, and parasitic pathogens in a single assay, overcoming the limitations of hypothesis-driven testing (Su et al., 2024). It provides a more actionable etiological pathway for complex or rare CNS infections and may facilitate earlier diagnosis and timely intervention (Ferreira et al., 2025). Meanwhile, given the latency/reactivation biology of betaherpesviruses, mNGS results should still be interpreted in the context of clinical consistency, CSF inflammatory evidence, and supportive findings from imaging and/or EEG.

According to the Infectious Diseases Society of America (IDSA), most HHV-6 infections are transient and asymptomatic and usually do not require treatment; however, when CNS involvement or other clinical syndromes occur, antiviral therapy should be initiated promptly (Tunkel et al., 2008). Because HHV-6 encephalitis in immunocompetent children is relatively rare, there is no universally accepted treatment regimen (Ward et al., 2019). The 2008 IDSA guideline recommends ganciclovir and/or foscarnet, although optimal dosing and duration remain unclear; some studies suggest at least 4 weeks of intravenous ganciclovir (Stathis et al., 2024; Tunkel et al., 2008).

In our study, all patients received antiviral therapy, but most courses lasted 1–3 weeks, possibly due to rapid clinical improvement, inter-individual variability, or drug-related adverse effects. In addition, foscarnet was not used in this cohort; regimens mainly involved ganciclovir or penciclovir/acyclovir, reflecting substantial heterogeneity in real-world practice. Overall short-term outcomes were favorable, but attention should still be paid to complications and post-infectious immune-related events.

Several limitations should be acknowledged. First, the small sample size and retrospective single-center design may limit the generalizability of our findings, and the results should be interpreted as descriptive rather than definitive comparisons between HHV-6 and HHV-7 encephalitis. Second, due to the retrospective design and the rarity of HHV-6/HHV-7 encephalitis in clinical practice, the sample size was limited, preventing a robust evaluation of the diagnostic performance of CSF mNGS. Finally, although HHV-6/HHV-7 attribution was based on mNGS findings combined with clinical and laboratory evidence, additional virological assessments, such as viral load quantification and longitudinal monitoring, were unavailable.

Additionally, chromosomally integrated HHV-6 (ciHHV-6) was not systematically excluded in this study due to the lack of quantitative viral load assessment and confirmatory testing from blood or hair/nail specimens. Therefore, the possibility of latent HHV-6 detection or reactivation cannot be completely excluded, which may limit definitive attribution of HHV-6 as the causative pathogen. Interpretation of HHV-6/HHV-7 mNGS findings should consider the potential effects of viral latency, reactivation, bystander detection, and background contamination.

## 5 Conclusions

This study characterized the clinical and metagenomic features of HHV-6 and HHV-7 encephalitis in immunocompetent children based on CSF mNGS combined with clinical evaluation. HHV-6 and HHV-7 encephalitis showed overlapping clinical and laboratory features. CSF mNGS provided complementary etiological information when interpreted together with clinical, CSF, imaging, and microbiological findings. Given the latent nature of HHV-6 and HHV-7, detection results should be interpreted cautiously.

## Data Availability

The data generated and analyzed in this study have been deposited in the EMBL-EBI European Nucleotide Archive (ENA) under accession number PRJEB127348. The dataset is publicly available at https://www.ebi.ac.uk/ena/browser/view/PRJEB127348.
